# Morphine and Clonidine Synergize to Ameliorate Low Back Pain in Mice

**DOI:** 10.1155/2012/150842

**Published:** 2012-04-23

**Authors:** Maral Tajerian, Magali Millecamps, Laura S. Stone

**Affiliations:** ^1^Alan Edwards Centre for Research on Pain, McGill University, Montreal, QC, Canada H3G 0G1; ^2^McGill Scoliosis & Spine Research Group, McGill University, Montreal, QC, Canada H3A 2B4; ^3^Department of Neurology & Neurosurgery, Faculty of Medicine, McGill University, Montreal, QC, Canada H3A 3R8; ^4^Faculty of Dentistry, McGill University, Montreal, QC, Canada H3G 0G1; ^5^Department of Anesthesia, Faculty of Medicine, McGill University, Montreal, QC, Canada H3G 1Y6; ^6^Department of Pharmacology & Therapeutics, Faculty of Medicine, McGill University, Montreal, QC, Canada H3G 1Y6

## Abstract

Chronic low back pain (LBP) is a debilitating condition associated with signs of axial and radiating pain. In humans with chronic LBP, opioids are often prescribed with varying outcomes and a multitude of side effects. Combination therapies, in which multiple pharmacological agents synergize to ameliorate pain without similar potentiation of adverse reactions, may be useful in improving therapeutic outcome in these patients. The SPARC-null mouse model of low back pain due to disc degeneration was used to assess the effects of opioid (morphine) and *α*_2_-adrenergic agonist (clonidine) coadministration on measures of axial and radiating pain. The results indicate that systemic morphine and clonidine, coadministered at a fixed dose of 100 : 1 (morphine : clonidine), show a synergistic interaction in reversing signs of axial LBP, in addition to improving the therapeutic window for radiating LBP. Furthermore, these improvements were observed in the absence of synergy in assays of motor function which are indicative of side effects such as sedation and motor incoordination. These data show that the addition of low-dose systemic clonidine improves therapeutic outcome in measures of both axial and radiating pain. Combination therapy could be of enormous benefit to patients suffering from chronic LBP.

## 1. Introduction

Low back pain (LBP) is a common condition associated with disability, decrease in quality of life, and significant economic burden [1–3]. Chronic LBP can include both axial and non-axial symptoms [4]. Axial LBP is characterized by spontaneous or movement-evoked pain or soreness confined to the spine and low back region. Radiating, non-axial LBP is pain that radiates from the back down one or both legs. This condition is often referred to as radicular pain or sciatica, because the pain usually follows the course of the sciatic nerve [5–8]. In animal models, radiating pain can be measured in the hindpaw. Although the exact mechanisms of LBP remain unclear, evidence suggests that the degeneration of intervertebral discs (IVDs) is associated with an increased risk of chronic LBP [9–12].

Pharmacotherapy is the most common treatment option for patients suffering from LBP with or without radiating pain [13]. Although non-steroidal anti-inflammatory drugs are the first line of defense against LBP, they do not sufficiently treat chronic and severe LBP. Opioids are often prescribed with varying therapeutic outcome [1, 14, 15] and are associated with undesired effects that limit their use, such as constipation, nausea, somnolence, fatigue, and the development of tolerance [16]. Since opioids such as morphine remain the gold standard of chronic pain treatment, it is vital to investigate strategies that would decrease required doses without diminishing the therapeutic effects. One such strategy is the addition of a non-opioid analgesic that will potentiate the analgesic effects of morphine without potentiating the undesirable adverse reactions.

The addition of *α*_2_-adrenergic agonists (*α*_2_ARs) improves opioid-induced antinociception in rodents following both systemic and spinal administration [17–25]. Evidence from human studies suggests that the use of opioid-*α*_2_AR agonist combinations in clinical pain management could minimize the side effects associated with both *α*_2_AR and opioid therapeutics [26, 27]. Furthermore, combination therapy may be effective in the treatment of chronic, opioid-insensitive pain states [28], and the *α*_2_AR agonist clonidine is approved for use in chronic pain. To date, the therapeutic benefit of opioid-*α*_2_AR agonist co-administration in chronic axial and non-axial LBP has not been systematically explored in either humans or animal models.

In this study, we used the SPARC-null mouse model of LBP due to disc degeneration (DD) to examine the effects of opioid-*α*_2_AR agonist combinations. SPARC (secreted protein, acidic, rich in cysteine; aka osteonectin or BM-40) is an evolutionarily conserved collagen-binding protein present in IVDs. SPARC is known to influence bone remodeling, collagen fibrillogenesis, and wound repair. Decreased expression of SPARC has been associated with aging and DD in human IVDs [29], and targeted deletion of the SPARC gene results in accelerated disc degeneration in the aging mouse [30]. DD in these mice is also associated with behavioural signs of axial and radiating LBP [31, 32].

The aim of the current study is to use the SPARC-null mouse model of low back pain to study the interaction between the prototypic opioid (morphine) and alpha-2 adrenergic agonists (clonidine) in treating signs of chronic axial and radiating pain.

Our results support the hypothesis that combination therapy using morphine and clonidine has the potential to improve therapeutic outcome for the chronic back pain patient.

## 2. Materials and Methods

### 2.1. Mice

SPARC-null mice (backcrossed to the C57BL/6 background) and wild-type (WT) controls (C57BL/6, Charles River, QC, Canada) were used as in previous studies [31–34].

4–6 month old male SPARC-null and WT control mice were bred in-house. Animals were housed in groups of 2–5, had unrestricted access to food and water, and were on a 12 hr light-dark cycle. All drug administration was adjusted for weight. SPARC-null mice were slightly lighter than WT mice (SPARC-null: 24.3 ± 0.3 at 4 months and 27.9 ± 0.4 at 6 months; WT: 25.9 ± 0.5 at 4 months and 32.1 ± 0.6 at 6 months). All experiments were performed blind to genotype and treatment, using a randomized block design.

All experiments were approved by the Animal Care Committee at the McGill University and conformed to the ethical guidelines of the Canadian Council on Animal Care and the guidelines of the Committee for Research and Ethical Issues of IASP [35].

### 2.2. Behavioural Analysis

#### 2.2.1. Tail Suspension Assay

Mice were suspended individually underneath a platform by the tail with adhesive tape attached 0.5 to 1 cm from the base of the tail and were videotaped for 180 s. The duration of time spent in (a) immobility (not moving but stretched out) and (b) escape behaviours (rearing to reach the underside of the platform, extending to reach the floor, or self-supported at the base of the tail or the suspension tape) were determined. The duration of immobility reflects the animal's willingness to stretch its main body axis. Deceased immobility is indicative of axial discomfort. This test is adapted from a traditional assay used to measure depression [36], and we have shown that it reliably measures signs of axial pain in mice [31, 32]. A cutoff of 180 seconds was applied when interpreting the data.

#### 2.2.2. Sensitivity to Cold Stimuli

A modified version of the acetone drop test was used [37], where the total duration of acetone-evoked behaviours (AEBs: flinching, licking, or biting) were measured in seconds for 1 minute after a drop of acetone (~25 *μ*L) was applied to the plantar surface of the hindpaw. An increased behavioural response to acetone suggests the development of cold allodynia and decreased reactivity is suggestive of antiallodynic efficacy. A cutoff of 4 s was applied when interpreting the data to facilitate isobolographic analysis.

#### 2.2.3. Rotarod Assay

 The accelerating rotarod assay was used to monitor animals for motor function (IITC Life Science Inc., Woodland Hills, CA, USA) with the mouse adapter (rod diameter, 3.2 cm) [38]. The task includes a speed ramp from 0 to 30 rotations per minute over 60 s, followed by an additional 240 s at the maximal speed. A decline in the latency to fall off the rotarod reflects motor incoordination. Mice were not trained prior to testing sessions. A cutoff of 200 s was used when interpreting the data.

#### 2.2.4. Open Field Assay

 A transparent open field apparatus (24 × 24 cm^2^) was placed in a quiet room illuminated with white light. The floor of the apparatus was equally divided into nine squares (8 × 8 cm^2^). Mice were individually placed into the open field on the central square, and their spontaneous behaviour was videotaped for 5 min. Subsequent analysis of the total number of squares visited was used to assess general motor activity [39]. An increase in the number of peripheral squares covered reflects hyperactivity, while a decrease is indicative of sedation. Following drug administration, animals underwent tail suspension just before being placed in the open field.

#### 2.2.5. Timeline

 The schedule of testing was as follows: 16 weeks of age: habituation to tail suspension; 20 weeks: baseline open field and tail suspension assays; 22 and 26 weeks: baseline and after drug administration for acetone and rotarod assays; 24 and 28 weeks: tail suspension and open field after drug administration. A wash-out period of 2 weeks was included between drug exposures to ensure that only the acute effects of each drug were studied.

### 2.3. Pharmacological Treatment

Analgesic agents or saline control were administered to SPARC-null and WT mice by i.p. injection (5 mL/kg injected directly in the intra-peritoneal cavity). Morphine (Medisca Inc., Montreal, QC, Canada) and clonidine (Sigma-Aldrich Canada Ltd., Oakville, ON, Canada) were dissolved in 0.9% saline either alone or in combination at a constant dose ratio of 100 : 1 (morphine : clonidine). Animals were tested 60 minutes after drug administration.

### 2.4. Data Analysis

#### 2.4.1. Behavioural Phenotype of LBP

Comparisons between saline-treated SPARC-null and WT mice were performed for each assay by 2-tailed, unpaired *t*-test. Welch's correction was used when the condition of equal variances was not met. Sample size ranged between 35 and 48 mice/group of saline-treated mice.

#### 2.4.2. Dose-Response Analysis (Table 1)

Individual dose points are reported as raw data for both strains and all pharmacological treatments as means with standard error of the mean (SEM). In order to calculate ED_50_ values, individual dose points were first converted to % maximum possible effect (%MPE) according to the following equations

(1) $$\begin{array}{cc} & \text{Tail}\;\text{suspension:}\\ & \text{\%}\;\text{MPE}=\frac{\text{drug}-\text{saline}}{\text{maximum}-\text{saline}}\times 100,\\ & \text{maximum}\;\text{effect}=180\;\text{seconds}\;\text{in}\;\text{immobility}. \end{array}\;$$

(2) $$\begin{array}{cc} & \text{Acetone:}\\ & \text{\%}\;\text{MPE}=\frac{\text{saline}-\text{drug}}{\text{saline}-\text{maximum}}\times 100,\\ & \text{maximum}\;\text{effect}=0\;\text{seconds}\;\text{of}\;\text{AEB-induced}\;\text{behaviour}. \end{array}\;\;$$

(3) $$\begin{array}{cc} & \text{Rotarod:}\\ & \text{\%}\;\text{MPE}=\frac{\text{saline}-\text{drug}}{\text{saline}-\text{maximum}}\times 100,\\ & \text{maximum}\;\text{effect}=0\;\text{seconds}\;\text{latency}\;\text{to}\;\text{fall}. \end{array}\;\;$$

(4) $$\begin{array}{cc} & \text{Open}\;\text{field:}\\ & \text{\%}\;\text{MPE}=\frac{\text{saline}-\text{drug}}{\text{saline}-\text{maximum}}\times 100,\\ & \text{maximum}\;\text{effect}=0\;\text{squares}\;\text{crossed}. \end{array}\;\;$$

ED_50_ values and confidence limits were calculated according to the graded dose-response method of Tallarida and Murray [40] on the linear portion of each dose-response curve. ED_50_ values were determined by extrapolation in cases where maximum efficacy was between 30 and 50%. If 30% efficacy was not reached, ED_50_ values were not calculated and was considered to lack efficacy. A minimum of three doses were used for each drug or combination of drugs.

#### 2.4.3. Isobolographic Analysis (Table 1)

Isobolographic analysis is the “gold standard” for evaluating drug interactions [40, 41]. Dose-response curves were constructed for each agonist administered alone, and the ED_50_ values were calculated. The two drugs were then coadministered at a constant dose-ratio approximately equal to their potency ratio, a third dose-response curve was constructed, and an experimentally derived combination ED_50_ was calculated.

To test for interactions between agonists, the ED_50_ values and standard error of all dose-response curves were arithmetically arranged around the ED_50_ value using the following equation: (ln⁡(10) × ED_50_) × (SEM  of  log⁡  ED_50_) [41]. Isobolographic analysis necessitates this manipulation. When testing an interaction between two drugs, a theoretical additive ED_50_ value is calculated for the combination based on the dose-response curves of each drug administered separately. This theoretical value is then compared by a *t*-test with the observed experimental ED_50_ value of the combination. An interaction is considered synergistic if the experimental ED_50_ is significantly less (*P* < 0.05) than the calculated theoretical additive ED_50_.

**Figure 1:**
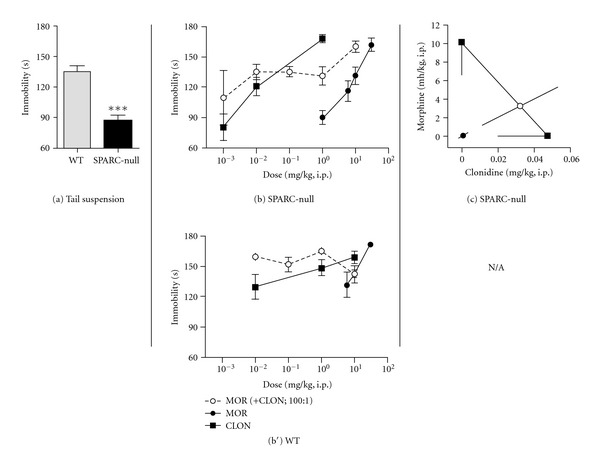
Morphine and clonidine synergize to attenuate axial pain in SPARC-null mice. (a) Saline-treated SPARC-null animals spend less time in immobility compared to WT mice in the tail suspension assay, indicative of axial pain. (b), (b′) In SPARC-null mice (b), morphine (●) and clonidine (■) dose-dependently inhibited axial pain when administered systemically either alone or coadministered (i.p.) at a constant dose ratio of 100 : 1 (morphine : clonidine). In WT mice (b′), morphine (●) and clonidine (■) dose-dependently inhibited axial pain when administered systemically, but the combination lacked efficacy. (c) Isobolographic analysis applied to the data from (b). The *y*-axis represents the ED_50_ for morphine, and the *x*-axis represents the ED_50_ for clonidine. The lines directed from each ED_50_ value toward zero are the lower 95% confidence limits of each ED_50_. The line connecting these two points is the theoretical additive line. The open circle on the theoretical additive line represents the calculated theoretical ED_50_ value of the combination if the interaction is additive. The observed combination ED_50_ (●) was significantly (*P* < 0.05; *t*-test) lower than the theoretical additive ED_50_ (∘), indicating that the interaction is synergistic. An isobolograph was not plotted for WT mice, since the combination lacked efficacy in this assay. Error bars represent ±SEM for each dose point (*n* = 5–11 animals/dose). See Table 1 for ED_50_ values. ***P < 0.0001.

**Figure 2:**
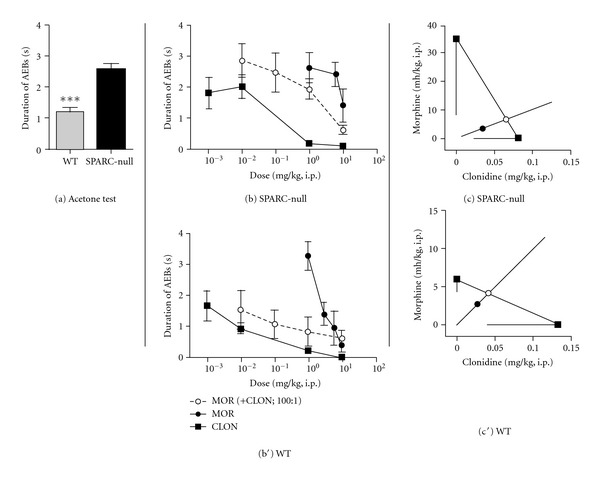
Effect of coadministration of morphine and clonidine on cold allodynia. (a) Saline-treated SPARC-null animals exhibit more acetone-evoked behaviours compared to WT mice in the acetone assay, indicative of cold hypersensitivity on the hindpaw. (b), (b′). In both SPARC-null (b) and WT (b′) mice, morphine (●) and clonidine (■) dose-dependently inhibited cold allodynia when administered systemically either alone or coadministered (i.p.) at a constant dose ratio of 100 : 1 (morphine : clonidine). (c), (c′) Isobolographic analysis applied to the data from (b), (b′). The *y*-axis represents the ED_50_ for morphine, and the *x*-axis represents the ED_50_ for clonidine. The lines directed from each ED_50_ value toward zero represent the respective lower 95% confidence limits of each ED_50_. The line connecting these two points is the theoretical additive line. The open circle on the theoretical additive line represents the calculated theoretical ED_50_ value of the combination if the interaction is additive. The observed combination ED_50_ (●) was not significantly different (*t*-test) from the theoretical additive ED_50_ (∘) in either strain, indicating that the interaction is additive in both cases. Error bars represent ±SEM for each dose point (*n* = 5–11 animals/dose). See Table 1 for ED_50_ values. ***P < 0.0001.

**Figure 3:**
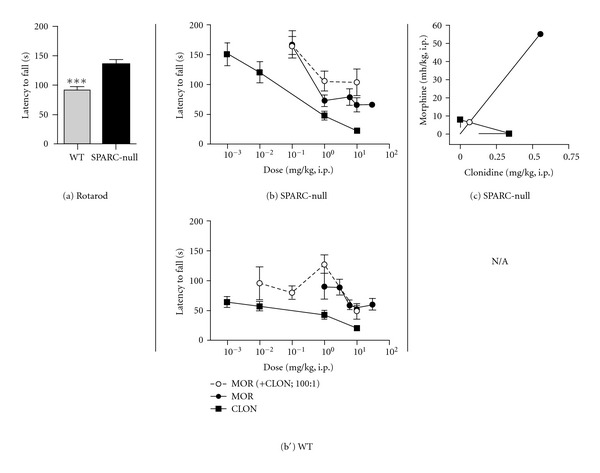
Effect of coadministration of morphine and clonidine on motor function. (a) Saline-treated SPARC-null animals perform better on the rotarod assay compared to WT mice, indicative of an absence of motor impairment in SPARC-null mice. (b), (b′) In SPARC-null mice (b), morphine (●) and clonidine (■) dose-dependently caused motor impairment when administered systemically either alone or coadministered (i.p.) at a constant dose ratio of 100 : 1 (morphine : clonidine). In WT mice (b′), morphine (●) and clonidine (■) dose-dependently caused motor incoordination when administered systemically, but the combination lacked efficacy. (c) Isobolographic analysis applied to the data from Figure 1(b). The *y*-axis represents the ED_50_ for morphine, and the *x*-axis represents the ED_50_ for clonidine. The lines directed from each ED_50_ value toward zero represent the respective lower 95% confidence limits of each ED_50_. The line connecting these two points is the theoretical additive line. The open circle on the theoretical additive line represents the calculated theoretical ED_50_ value of the combination if the interaction is additive. The observed combination ED_50_ (●) was not significantly different (*t*-test) from the theoretical additive ED_50_ (∘), indicating that the interaction is additive. Isobolographic analysis was not performed in WT mice since the combination lacked efficacy in this assay. Error bars represent ±SEM for each dose point (*n* = 5–11 animals/dose). See Table 1 for ED_50_ values. ***P < 0.0001.

Visualization of drug interactions can be facilitated and enhanced by graphical representation of isobolographic analysis (Figures 1, 2, and 3, c–c′). This representation depicts the ED_50_ of each agent on the *x*- or *y*-axis. For example, Figure 1(c) presents the ED_50_ of morphine on the *y*-axis and the ED_50_ of clonidine on the *x*-axis. The line connecting these two points depicts the dose combinations expected to yield 50% efficacy if the interaction is purely additive and is called the theoretical additive line. The theoretical additive ED_50_ and its confidence interval are determined mathematically and plotted spanning this line. The observed ED_50_ for the combination is plotted at the corresponding *x*, *y* coordinates along with its 95% confidence interval for comparison to the theoretical additive ED_50_. Isobolographs were plotted only when both drugs alone and the combination showed efficacy.

All dose-response and isobolographic analyses were performed with the FlashCalc pharmacological statistics software package generously supplied by Dr. Michael Ossipov.

#### 2.4.4. Therapeutic Window (Table 2)

Therapeutic window (TW) is a measure of the amount of an agent required to produce the desired effect (i.e., analgesia) compared to the amount that produces the undesired effect (i.e., motor impairment). In this study we define the TW as the ED_50_ (undesired effect)/ED_50_ (desired effect). A TW < 1 indicates the drug is more potent in the production of the undesired effect than the desired effect. A TW > 1 indicates that the desired effect can be achieved in the absence of the side effect. Higher indices are more advantageous therapeutically.

## 3. Results

### 3.1. Morphine and Clonidine Synergize to Improve Axial Pain in the Tail Suspension Assay

SPARC-null mice show signs of axial pain compared to WT mice as shown in the tail suspension assay (135.4 ± 5.2 s in WT versus 86.8 ± 5.7 s in SPARC-null, *P* < 0.0001, 2-tailed *t*-test, Figure 1(a)). Both in SPARC-null and WT mice, systemic administration of either morphine or clonidine produced dose-dependent increases in immobility, indicative of reduced axial discomfort, 60 minutes after injection (Figures 1(b), 1(b′)).

The dose-response data from Figure 1(b) is represented graphically as an isobologram in Figure 1(c). As shown in Figure 1(c), the ED_50_ of the combination (closed circle) in SPARC-null mice is lower than the theoretical additive ED_50_ (open circle), indicating that this interaction is synergistic. This synergistic interaction was confirmed by statistical comparison between the observed combined ED_50_ value and the theoretical additive ED_50_ value.

In WT mice, all morphine + clonidine coadministration doses showed similar efficacy in the range tested (Figure 1(b′)). Additional doses of this combination need to be explored to resolve the dose-response relationship necessary for isobolographic analysis (Table 1).

### 3.2. Morphine and Clonidine Are Additive in the Acetone Test of Cold Allodynia

SPARC-null mice show signs of cold allodynia on the hindpaw compared to WT mice, as shown in the acetone assay (1.2 ± 0.1 s in WT versus 2.6 ± 0.2 s in SPARC-null, *P* < 0.0001, 2-tailed *t*-test, Figure 2(a)). In SPARC-null mice, systemic administration of clonidine produced dose-dependent analgesia in the acetone assay at 60 minutes after injection, while morphine failed to reach 50% MPE but was of sufficient maximum efficacy (45%) to extrapolate an ED_50_ value (Figure 2(b)).

In WT mice, the administration of either morphine or clonidine alone produced dose-dependent antinociception in the acetone assay (Figure 2(b′)). This interaction was tested statistically by comparing the observed combined ED_50_ value and the theoretical additive ED_50_ value and was shown to be additive. The dose-response data from Figures 2(b), 2(b′) are represented graphically as isobolograms in Figures 2(c), 2(c′). As shown in Figures 2(c), 2(c′), the ED_50_ of the combination (closed circle) in both strains is not significantly different from the theoretical additive ED_50_ (open circle), indicating that this interaction is additive (Table 1).

### 3.3. Morphine and Clonidine Are Additive in the Rotarod Test of Motor Impairment

SPARC-null mice do not show signs of motor impairment at 6 months of age. Rather, they perform better than WT mice in the rotarod assay at this age (92.1 ± 5.9 s in WT versus 136.2 ± 6.2  s in SPARC-null, *P* < 0.0001, 2-tailed *t*-test, Figure 3(a)). In SPARC-null mice, systemic administration of either morphine or clonidine produced dose-dependent motor impairment in the rotarod assay at 60 minutes after injection (Figure 3(b)). Only clonidine produced a dose-dependent effect in WTs (Figure 3(b′)).

The SPARC-null dose-response data from Figure 3(b) is represented graphically as an isobologram in Figure 3(c). As shown in Figure 3(c), the ED_50_ of the combination (closed circle) is not significantly different from the theoretical additive ED_50_ (open circle), indicating that this interaction is additive. In WT mice, morphine + clonidine coadministration lacked efficacy, and thus it was not possible to perform isobolographic analysis in this assay (Table 1).

### 3.4. Opposing Effects of Morphine and Clonidine in the Open Field Test of Voluntary Activity

**Figure 4:**
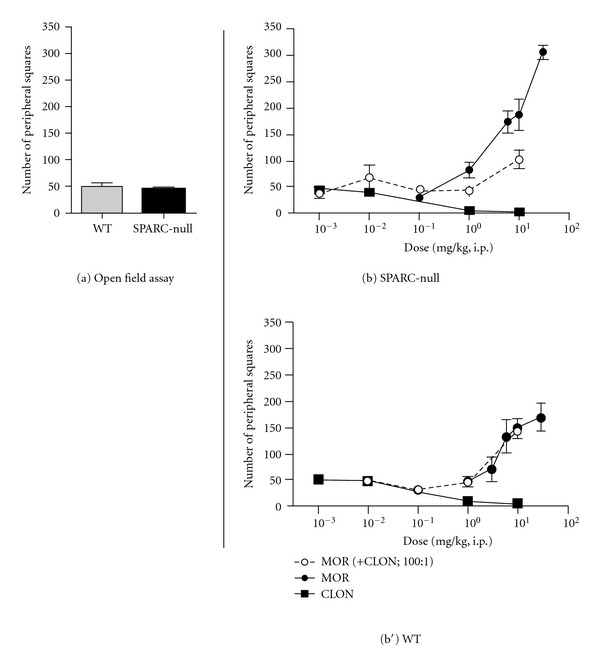
Effect of coadministration of morphine and clonidine on overall activity. (a) Saline-treated SPARC-null animals do not differ from WT mice in the number of peripheral squares covered in the open field, indicative of comparable overall activity between the two strains. (b), (b′). Both in SPARC-null (b) and WT (b′) mice, morphine (●) caused an increase in activity, while clonidine (■) dose-dependently caused sedation. When coadministered (i.p.) at a constant dose ratio of 100 : 1 (morphine : clonidine), the combination showed no efficacy in SPARC-null mice and produced hyperactivity in WT mice. No isobolographs were plotted for either strain as the drugs had opposing effects. Error bars represent ±SEM for each dose point (*n* = 5–11 animals/dose). See Table 1 for ED_50_ values.

SPARC-null mice do not differ from WTs in the number of peripheral squares covered in the open field, indicative of no overall change motor activity (49.1 ± 5.9 squares in WT versus 45.7 ± 3.8 squares in SPARC-null, *P* = 0.6, 2-tailed *t*-test, Figure 4(a)). In both SPARC-null and WT mice, systemic administration of morphine produced dose-dependent hyperactivity, while clonidine produced dose-dependent sedation in the open field assay at 60 minutes after injection in (Figure 4(b), 4(b′)). Since the two agonists exert opposite effects on overall activity, isobolographic analysis was not performed for the open field test.

### 3.5. Effect of Morphine and Clonidine Coadministration on Therapeutic Window

The data presented above demonstrate that coadministration of morphine and clonidine produces antinociceptive but not sedative synergy following i.p. administration. We therefore examined the impact of coadministration on the therapeutic window (TW) between sedation and antinociception. In Table 2, the TW has been calculated for morphine and clonidine alone and in combination following systemic administration for both axial pain and cold allodynia. In SPARC-null mice, the window for each drug given alone ranged from 0.2 and 6.8, indicating little separation between the antinociceptive and sedative effective dose ranges. In contrast, the addition of a small amount of clonidine to morphine increased these values to 700 for axial LBP and 16 for non-axial LBP. These changes reflect the fact that analgesia is reached before sedation when the drugs are coadministered. These increases in therapeutic window are the result of potentiation in the antinociceptive assays in parallel with an additive interaction in the undesired side effect (motor impairment).

## 4. Discussion

### 4.1. Morphine and Clonidine Synergy Improves Therapeutic Outcome for Axial Pain

SPARC-null mice develop behavioural signs of axial pain by 4–6 months of age concurrent with disc degeneration [31, 32, 42]. In the current study, we show that while morphine and clonidine dose-dependently attenuate axial pain, the side effects of motor impairment, sedation (clonidine), and hyperactivity (morphine) develop in a similar dose range. Systemic coadministration of morphine and clonidine not only resulted in synergy in SPARC-null but also the therapeutic window of the combination was greater than for either drug administered alone. The pharmacological effects observed in SPARC-null animals are not likely due to motor impairment or sedation, since the morphine + clonidine combination lacked efficacy in our tests of motor function. Furthermore, while morphine produced increases in overall activity, morphine-treated animals spent more time in immobility in the tail suspension assay, indicative of antinociception.

The majority of preclinical studies examining opioid-*α*_2_AR interactions to date have been carried out in naïve rodents, where the measured endpoint is antinociception to cutaneous noxious stimuli [21–25, 43] or inhibition of chemically-evoked behaviours [44, 45]. In contrast, the current study focused on pharmacological reversal of pathological signs of axial LBP in a preclinical model of intervertebral disc degeneration-related pain. To our knowledge this is the first demonstration of an opioid-adrenergic antinociceptive synergy in LBP in preclinical studies.

In patients suffering from axial LBP, pain management remains inadequate. Patients with mild or severe LBP are often prescribed two or more medications in addition to opioids, reflecting the challenging nature of LBP [46]. Currently the primary use of clonidine as a pain management tool is as a spinal adjuvant for opioids in intractable cancer pain [47]. Although not currently indicated for patients with chronic axial LBP, our results suggest that low doses of systemic clonidine may be a useful addition to opioid therapy.

### 4.2. Coadministration of Morphine and Clonidine Increases the Therapeutic Window for Radiating Pain

Cold allodynia in the hindpaw of SPARC-null mice is a behavioural measure of non-axial, radiating pain. While cold allodynia is reversed by systemic clonidine, that efficacy is associated with side effects including motor impairment and sedation. Although the coadministration of morphine and clonidine was additive in our model, we did observe an improvement in the therapeutic window, such that therapeutic effects were observed at doses associated with minimal side effects. We therefore believe that suppression of cold allodynia by the combination of morphine and clonidine is independent of motor impairment.

Radiating pain, which may accompany axial pain in patients suffering from LBP [5–8], is thought to have a mainly neuropathic mechanism [48]. As a result, anti-neuropathic agents and not opioids are the treatment of choice in these patients. Consistent with the reduced opioid efficacy commonly associated with neuropathic pain conditions, morphine failed to reach 50% efficacy in cold hypersensitivity in SPARC-null mice in the current study. Furthermore, while the ED_50_ values for morphine were between 8 and 10 mg/kg in the tail suspension and rotarod assays, the extrapolated ED_50_ value for morphine in non-axial pain was >30 mg/kg. These observations support the predictive validity of the current model.

Studies evaluating opioid-*α*_2_AR agonist interactions in rodent models of neuropathic pain have demonstrated synergistic interactions between morphine and the *α*_2_AR agonists clonidine and moxonidine [17, 49]. While morphine and clonidine coadministration did not result in synergy in radiating pain in the current study, it did improve the therapeutic window in this modality. Previous work demonstrating that opioid-*α*_2_AR synergy is sensitive to both route of administration and the behavioral endpoint could explain this seeming discrepancy [22], as could the use of chronic pain models with different etiologies.

These results, together with the synergy observed in axial analgesia, demonstrate that combinations of morphine and clonidine target both the axial and radiating pain aspects observed in SPARC-null mice. In humans, the ability to obtain sufficient relief of both axial and radiating pain with the combination of morphine and a low dose of clonidine could result in less adverse drug reactions, fewer undesired or unanticipated drug interactions, increased patient compliance, and improved quality of life.

### 4.3. Opioid-*α*_2_AR Agonist Interactions

In humans, only a few studies have examined the interaction between opioid-*α*_2_AR agonists in chronic pain conditions. In one study, the addition of epidural clonidine benefited patients with intractable cancer pain, particularly those with a significant neuropathic component [47], and the combination of intrathecal morphine + clonidine is useful for the management of chronic pain after spinal cord injury [50, 51]. In order to maximize the clinical relevance of the current study, systemic administration was selected; spinal delivery requires invasive procedures that add additional risks. A variety of systemically delivered adrenergic agonists (i.e., clonidine, dexmedetomidine, moxonidine, tizanidine) are currently available for use in humans and could be utilized as adjuvants in patients not receiving sufficient efficacy from opioids.

Although there are many studies reporting functional interactions between opioids and *α*_2_AR agonists (for review see [52]), the molecular mechanisms underlying these interactions are not clear. Depending on the agonists used, analgesic synergy may be mediated by *α*_2A_-, *α*_2B_-, or *α*_2C_-adrenergic receptor subtypes and mu- or delta-opioid receptors [44, 53–55]. Evidence from immunohistochemical studies suggests that opioid receptors are coexpressed in the same population of sensory neurons as *α*_2_ARs [56] and that antinociceptive synergy requires activation of calcium channels [57, 58] and protein kinase C [45, 59]. Physical association between G protein-coupled receptors such as the opioid and adrenergic receptors has been proposed to account for the synergistic effects observed [56, 60, 61]. It is well established that coexpression of GPCRs results in the formation of heteromeric complexes with altered functional and ligand binding properties [62]. Such interactions could occur at the level of the primary afferent neurons, the spinal cord and other sites in the CNS (i.e., locus coeruleus [63]), as well as in the periphery.

## 5. Future Directions

We have studied the acute effects of morphine, clonidine, and their combination 60 minutes after systemic administration. However, in clinical situations most patients undergo chronic pharmacotherapy. It is therefore critical to study these interactions using a chronic dosing paradigm. The use of multimodal therapy may be of even greater therapeutic benefit if chronic studies reveal protective effects of the combination against the development of tolerance or opioid-induced hyperalgesia. Clonidine is also known to reduce opioid withdrawal symptoms, a property that may be beneficial in long-term management of chronic noncancer pain [64].

Our study was carried out in a transgenic mouse model of LBP due to disc degeneration. While this model incorporates pharmacologically reversible behavioral measures of both axial and radiating pain associated with progressive, age-dependent intervertebral disc degeneration [31, 32, 42], it is unlikely to fully parallel patients suffering from LBP. Ultimately further studies in both preclinical models and human subjects are required to fully understand the therapeutic benefit of adrenergic adjuvant therapy.

## 6. Conclusions

We have used a mouse model of chronic LBP due to progressive disc degeneration to explore the effects of morphine and clonidine coadministration on measures of axial and radiating pain. Side effects including motor impairment and overall change in activity were also assessed. This is the first study to report a synergistic interaction between clinically used analgesics in a rodent model of chronic low back pain and to include the measurement of both axial and radiating pain. The results indicate that the addition of low-dose systemic clonidine can improve therapeutic outcomes both in axial and radiating pain measures, which could be of enormous benefit to patients suffering from chronic LBP.

## Figures and Tables

**Table 1:** Effect of combination therapy on drug potency.

Assay	Strain	Morphine ED_50_	Clonidine ED_50_	Observed	Theoretical	Interaction
				combination	combination	
				ED_50_	ED_50_	
Tail suspension (Axial pain)	SPARC-null	10 (±4.0)	0.05 (±0.04)	0.08 (±0.23)	3.3 (±2.1)	**Synergistic**
	WT	18 (±6.0)	8.2 (±21)	NA	17 (±5.7)	NA

Acetone (cold allodynia)	SPARC-null	*~35 (±50)*	0.08 (±0.09)	3.5 (±6.3)	6.6 (±6.0)	**Additive**
	WT	6 (±2.0)	0.1 (±0.2)	2.7 (±8.9)	4.2 (±1.8)	**Additive**

Rotarod (motor incoordination)	SPARC-null	8 (±6.1)	0.3 (±0.3)	*~56 (±85)*	6.5 (±4.5)	**Additive**
	WT	*~17 (±14)*	0.1(±0.2)	No efficacy	7.3 (±7.4)	NA

Open field (overall activity)	SPARC-null	*0.2 (±0.1)	0.2 (±0.2)	No efficacy	NA	NA
	WT	*0.6 (±0.3)	0.14(± 0.16)	*0.13 (± 0.08)	NA	NA

Morphine and clonidine ED_50_ values (mg/kg, i.p.) either alone or in combination at a dose ratio of 100 : 1 (observed combination ED_50_). The Theoretical Combination ED_50_ is the predicted ED_50_ for the combination in the absence of any interaction. The interaction indicates if the observed combination ED_50_ was statistically different from the theoretical combination ED_50_. ~ indicates that the ED_50_ value was determined by extrapolation if maximum efficacy was less than 50%. *In the open field assay, morphine had no potency as a sedative but caused hyperactivity. A drug or drug combination was considered to exhibit no efficacy if maximum efficacy was under 30%. NA = not available (it is not possible to calculate these values when one drug lacks efficacy).

**Table 2:** Combination therapy improves therapeutic window.

Strain	Drug(s)	ED_50 _ value (±SE; mg/kg, i.p.)			Therapeutic window	
		Motor	Axial	Non-axial	Motor/axial	Motor/non-axial
SPARC-null	Morphine	8 (±6.1)	10 (±4.0)	*~35 (±50)*	0.8	0.2
	Clonidine	0.3 (±0.3)	0.05 (±0.04)	0.08 (±0.09)	6.8	4.3
	Morphine (+ CLON; 100 : 1)	*~56 (±85)*	0.08 (±0.23)	3.5 (±6.3)	**700**	**16**

WT	Morphine	*~17 (±14)*	18 (±6.0)	6.0 (±2.0)	0.9	2.8
	Clonidine	0.1 (±0.2)	8.2 (±21)	0.1 (±0.2)	0.01	1.0
	Morphine (+ CLON; 100 : 1)	*No efficacy*	*No efficacy*	2.7 (±8.9)	N/A	N/A

The Therapeutic window is the ratio of the ED_50_ value (mg/kg, i.p.) of the undesired effect (motor impairment) to the desired effect (inhibition of axial or non-axial pain). A larger therapeutic window suggests the drug or drug combination will be analgesic at doses that do not produce motor impairment. ~ indicates that the ED_50_ value was determined by extrapolation if maximum efficacy was less than 50%. NA = not available (the combination lacked efficacy in the rotarod assay in WT mice). Note the much larger therapeutic window achieved with the addition of clonidine to morphine.
